# Genotoxic Effects of Exposure to Water-Soluble Fraction of Diesel Fuel in Sand Dollar *Scaphechinus mirabilis* Gametes

**DOI:** 10.3390/toxics11010029

**Published:** 2022-12-28

**Authors:** Victor Pavlovich Chelomin, Valentina Vladimirovna Slobodskova, Sergey Petrovich Kukla, Elena Vladimirovna Zhuravel, Andrey Pavlovich Chernyaev

**Affiliations:** 1 Il’ichev Pacific Oceanological Institute, Far Eastern Branch, Russian Academy of Sciences, 690041 Vladivostok, Russia; 2 International UNESCO Department of Marine Ecology, Institute of the World Ocean, Far Eastern Federal University, 690922 Vladivostok, Russia; 3 Institute of High Technologies and Advanced Materials, Department of Chemistry and Materials, Far Eastern Federal University, 690922 Vladivostok, Russia

**Keywords:** petroleum hydrocarbons, genotoxicity, *S. mirabilis*, comet assay

## Abstract

Pollution of marine areas with oil and oil products is steadily growing. As part of this connection, the study of the impact of petroleum hydrocarbons on marine hydrobionts is an urgent issue of modern ecotoxicology. In our study, the genotoxic effect of the water-soluble fraction of diesel fuel at different concentrations on the gametes of the sand dollar *Scaphechinus mirabilis* was evaluated. It was shown that during the incubation of sperm and eggs of a sand dollar in WAF with an oil hydrocarbon content of 1.32; 2.64; 5.37; 7.92 mg/L caused the destruction of the DNA molecule to varying degrees in both types of gametes. In addition, it has been shown that with an increase in the concentration of petroleum hydrocarbons in WAF, a large number of cells with a high level of DNA damage appear. The success of fertilization after exposure of gametes to a water-soluble extract of petroleum hydrocarbons was also evaluated. The relationship between an increase in the concentration of hydrocarbons in the tested solutions and a decrease in the level of fertilization is shown.

## 1. Introduction

Oil and its products constantly attract increased attention from ecotoxicologists, due to their potential harmful effects on the environment. The consequences of exposure to petroleum hydrocarbons are especially dangerous for aquatic ecosystems, since in the modern world a significant amount of oil is produced offshore, and the main transportation is carried out by tanker fleets across the world’s oceans. In spite of strict maritime safety regulations, the number of oil and petroleum product spills is very high, and on a global scale, is steadily increasing due to the increasing demand for oil [1,2]. Terrigenous runoff makes a significant contribution to the pollution of coastal waters of the seas. Today, petroleum hydrocarbons are still the main pollutant in coastal waters, especially in semi-enclosed bays with a slowed water exchange.

Due to the increasing level of oil pollution in the marine environment, studying the effects of oil hydrocarbons on marine organisms is becoming increasingly important. Most studies have focused on the toxic effects of oil on various aquatic organisms, including marine benthic invertebrates that absorb these contaminants from the water column and sediments [3].

Although petroleum hydrocarbons affect aquatic organisms as a physical factor through changes in habitat (reduction in light, availability of food and dissolved oxygen, increase in viscosity, etc.), the fraction responsible for toxic effects is the water-soluble fraction of petroleum hydrocarbons (WSF). After oil products enter the marine environment, some of them—mainly low molecular weight components—are quickly removed by evaporation, but most of the remaining oil exists in the form of a WSF. WSF is believed to be saturated with petroleum products, mainly hydrocarbons, which may be in a true solution or as a stable emulsion [4].

In general, numerous studies have shown that the WSF of various types of oils and petroleum products is toxic to a wide range of aquatic organisms, causing a multiple range of adverse biological effects that have negative consequences for natural resources. For example, damage to the DNA of hemocytes and cells of the digestive gland was noted in the bivalves *Mytilus edulis* and *Mya arenaria*, while in the amphipods *Quadrivisio aff. lutzi*, in cells of the gills of *Corbicula fluminea*, a decrease in the success of fertilization when exposed to *Nereis virens* oocytes was shown, and the inhibition of antioxidant defense was observed in juvenile *Gadus morhua*, polychaete *Laeonereis culveri* and the bivalve *Anomalocardia flexuosa* [5,6,7,8,9,10,11,12,13].

In modern ecotoxicology, the early stages of development of various species of marine invertebrates, particularly sea urchins, are among the most common biological models used to test the toxicity of various types of pollution. This is due to the point of view generally accepted in the literature that gametes, embryos and larvae are more sensitive than adults, and represent a critical period in the life cycle of an organism [14,15].

During any spill event, the resulting WSF is highly likely to affect the reproductive performance of marine invertebrates, as gametes (oocytes and spermatozoa), developing embryos and larval stages are freely released into the water column. Additionally, gametes and larvae are part of the plankton, and unlike actively swimming organisms, are not able to avoid polluted water masses. Therefore, egg fertilization success and larval development are widely used as tests for marine toxicity. WSF has been shown to be highly toxic to the early stages of development of various aquatic organisms, including fish, crustaceans, mollusks and sea urchins [3,12,16]. In addition to lethal consequences, petroleum water-soluble hydrocarbons caused structural deformations, developmental anomalies and various biochemical changes in the early life stages of marine organisms.

When any biochemical or structural damage affects gametes and embryos, the result can be impaired fertility, which subsequently affects the rate of reproduction and health of the offspring, and leads to significant effects at the population level [17,18,19,20]. Therefore, the study of changes in gametes at the molecular level could predict the consequences for reproductive success at an early stage, linking these consequences to the next generation.

Currently, in ecotoxicological studies, approaches that reveal the genotoxic effect of pollutants are gaining popularity. Among the many molecular biochemical processes, interest in genetic damage is increasing every day, mainly due to the fact that DNA is one of the main targets when exposed to stress factors [21]. Altered DNA can initiate a cascade of adverse changes, with serious consequences ranging from possible mutations to cell death [22].

The nuclear DNA of germ cells is a critical target for the main reason: DNA controls the physiological and biochemical processes of early development, and disruption of DNA integrity can later affect most life processes, including fertilization, cleavage, organ differentiation, formation, etc. In this regard, however, the influence of the water-soluble fraction of petroleum hydrocarbons on the genetic integrity of the gametes of marine invertebrates has been poorly studied.

Based on the foregoing, the aim of this work was to investigate the potential risk of exposure to petroleum hydrocarbons, using the example of a water-soluble fraction of diesel fuel (WAF-DF) present in diesel fuel, on the integrity of gamete DNA, with a subsequent effect on their ability to fertilize.

In our work, we used the Comet assay, which is widely applied in ecotoxicological studies as a sensitive tool for assessing DNA damage in individual eukaryotic cells [23]. Experts estimate that this molecular approach is dozens of times (35 to 50 times) more sensitive than any biomarker used to assess the degree of toxicity at the organism level (such as survival, reproduction, growth, etc.) [24].

## 2. Material and Methods

### 2.1. Elutriate Preparation and Analyses

For the elutriate preparation, diesel fuel (ASTM D975) was mixed with control filtered seawater in a proportion of 1:10 in a 2 L glass bottle, and intensively stirred for 1 h. Then, the mixture was left for 16 h to allow the separation of phases. The liquid phase containing the water accommodated hydrocarbons (WAF-DF) was removed by siphoning and filtering through paper “white tape”, and used for toxicity testing and chemical analyses.

The total amount of hydrocarbons in the elutriate was revealed using an IR analyzer from Bruker Vertex 70 (USA). Total hydrocarbons were determined in the form of their solution in hydrocarbon tetrachloride, according to a pre-built calibration curve from 0 to 10 mg/L.

To determine the content of polyaromatic hydrocarbons (PAH), reverse-phase high-performance liquid chromatography is used. The analysis is carried out on a liquid chromatograph LC-20 Prominence (Shimadzu, Japan),. and the detector is a fluorescent RF—10Axl (Shimadzu, Japan). Chromatographic column—PAH C18 (25 cm*0.46 cm, 5 µm), (Waters, USA) A mixture of acetonitrile (Kriohrom, Russia) and distilled water is used as the eluent in the gradient elution mode. The eluent feed rate is 1 mL/min. The PAH mix 14 of Sigma-Aldrich (St. Louis, MO, USA) was used as a reference material. The analyses were performed in accordance with the patent of the Russian Federation for the preparation and extraction of samples for the floresil Sigma-Aldrich (St. Louis, MO, USA column [25]. Concentrations of calibration solutions of polyaromatic hydrocarbons were in the range of 1 to 200 μg/mL.

For the quantitative determination of petroleum hydrocarbons by gas chromatography, the Agilent 6890 gas chromatograph (Santa Clara, MO, USA) with a flame ionization detector was used. After extraction with hexane, the exact amount of 1,3,5-triphenylbenzene was added to the resultant solution as an internal standard. As a test mixture for calibration dependences, we used the Alkane standard mixture for performance tests of GC-systems (Supelco Merck Life Science LLC Valovaya Str. 35, floor 6 Moscow 115054 Russian Federation).

For the toxicity testing, elutriate was diluted with control seawater to obtain solutions of 12.5, 25, 50 and 75% of elutriate. A bioassay was conducted with four dilutions. A bioassay was conducted with four dilutions (1.32, 2.64, 5.37, 7.92 mg/L).

### 2.2. Experimental Procedure

The experiment was carried out at the marine experimental station “Popov Island” Il’ichev Pacific Oceanological Institute. The sand dollars *Scaphechinus mirabilis* (Agassiz, 1864) were collected by SCUBA divers from natural populations in the area with low anthropogenic impact in the Peter the Great Bay at a depth of 4–4.5 m. They were acclimated in aerated seawater for 1 week. The water temperature during the experiments was maintained at 17–18 °C, and water salinity at 32.2–32.6‰.

Spawning was induced by injection of 0.2 mL, 0.5 M KCl (Helicon, Moscow, Russia) solution into the body cavity. Eggs were obtained in 50-mL beakers filled with sea water, which were rinsed with fresh sea water several times, decanted and used within 1 h at the latest [26,27]. Sperm cells were collected immediately before the experiment. Prior to the fertilization, gamete viability was assessed by checking under the microscope egg roundness and sperm motility. Then, the level of egg fertilization in fresh seawater was checked. Eggs with a level of fertilization below 95% were not used in the experiment. As a result, three male and female individuals were selected for the experiment. For each bioassay, data quality was assessed by a negative control test with natural seawater, filtered through a three-fraction gravel filter and sterilized by ultraviolet radiation.

Three types of experiments were conducted. In the first, the sperm cells were incubated for 1 h in elutriates. To achieve fertilization, the egg cells were placed as a monolayer into a beaker with 40 mL of sterile sea water, and 0.2 mL of incubated sperm cells were added; diluted 20,000 times (the final dilution was 40,000–60,000 times). In the second experiment, eggs were incubated in elutriates for 1 h, and were then replaced in the beaker with 40 mL of control water and fertilized with unexposed sperm. In the third experiment, both eggs and sperm cells were incubated in elutriates for 1 h after the incubation fertilization was conducted.

### 2.3. Comet Assay

Genotoxicity is assessed using the alkaline comet assay [28], which is adapted to marine organisms [29]. 50 μL of cell suspension was added to 100 μL of 1% low-melting agarose (MP Biomedicals, Eschwege, Germany) in 0.04 M phosphate buffer (pH 7.4) at 37 °C, carefully changed, applied to a glass slide, pre-coated for better adhesion with 1% solution agarose and covered with a coverslip. The sample was placed in a refrigerator for 3 min to gel. The coverslip was carefully removed and immersed in a lysis solution (2.5 M NaCl; 0.1 M EDTA-Na_2_; 1% Triton X-100; 10% DMSO; 0.02 Ms, pH 10) for 1 h in a dark, cold place. After washing with distilled water, the slides were placed in electrophoresis buffer (300 mM NaOH, 1 mM EDTA-Na_2_) and kept for 40 min. Electrophoresis is carried out at a voltage of 2 V/cm for 15 min. After neutralization (0.4 M Tris-HCl, pH 7.4), the slides are stained with ethidium bromide (2 μg/mL).

Visualization and registration of DNA comets is carried out using a scanning fluorescence microscope (Zeiss, AxioImager A1), equipped with an AxioCam MRc digital camera. To process digital images, we used the selective comet processing method proposed by Collins et al. [30]. This approach involves the division of comets into 5 classes (C0, C1, C2, C3 and C4), according to the degree of damage. Based on this classification, a genetic damage index (GDI) was calculated using the formula [C1 + (2 × C2) + (3 × C3) + (4 × C4)]/(C0 + C1 + C2 + C3 + C4), where C0, C1, C2, C3 and C4 are the number of respective class comets [31]. In each experimental group, 5 slides were analyzed (1 slide = 1 individual), containing two coverslips with at least 50 comets in each.

### 2.4. Statistical Analysis

The results of the experiments were processed in MS Excel and Statistica software packages; the arithmetic mean and standard deviation were calculated. The significance of the differences in comet assay and fertilization between the control and experimental groups was assessed by means of a one-factor analysis of variance using Dunnett’s test (at *p* < 0.05).

## 3. Results and Discussion

One of the main sources of oil hydrocarbons entering the coastal waters is maritime transport, which operates mainly on diesel fuel and fuel oil. For example, 45% of petroleum hydrocarbons in US coastal waters come from diesel fuel [32]. Mainly aliphatic and aromatic compounds of medium (intermediate) size and hydrocarbons that have undergone photodegradation during weathering pass into the aqueous phase. The composition of hydrocarbons that have passed into the water phase largely depends on the chemical characteristics of the spilled oil products, and is determined by the varying conditions of the marine environment. Therefore, it is very difficult to fully reproduce this process in laboratory conditions. However, certain steps in the standardization of laboratory methods for the extraction of petroleum hydrocarbons and the production of WAF have allowed this approach to be widely accepted among ecotoxicologists, especially in comparative studies of WAF toxicity and sensitivity of various species of marine organisms [3,4,5,6,7,8,9,11,13].

Like many other chemicals, the aqueous phase hydrocarbons (WAF) of petroleum were far more toxic to early life stages than to adults. Previous studies have shown that most marine gamete, embryonic and larval species were sensitive to WAF, as their LC50 ranges ranged from 53 µg/L to 13 mg/L, depending on the animal species and composition of WAF [3,8].

Considering that the gametes of marine invertebrates are released directly into sea water and are unprotected, there remains a high probability of a direct effect of dissolved petroleum hydrocarbons on the processes of fertilization and development. Furthermore, gametes and larvae are part of plankton, and unlike actively swimming organisms, are not able to avoid polluted water masses. Therefore, bivalve and sea urchin embryos and larvae are among the most common biological models used to study the effects of WAF oil on aquatic reproduction [7,16,31,32].

The sand dollar *S. mirabilis* proposed as an object of study is widely distributed in the Far East region, and is well studied and sensitive to the impact of natural and anthropogenic factors [33,34,35]. The sand dollar *S. mirabilis*, as a typical representative of the class of sea urchins, throws out germ cells directly into sea water during the spawning period, where fertilization and further development of embryos and larvae take place. Directly in sea water, the outer membranes and receptors of gametes and embryos of the early stages of development of the sand dollar are directly exposed to various adverse factors, including the WAF. In this regard, our laboratory experiments on the interaction of WAF with sea urchin gametes to a certain extent imitate the real situation that occurs when oil or oil products enter the marine environment.

Diesel fuel is a very complex mixture of hydrocarbons. According to the results of chromato-mass-spectrometric analysis (Table 1), the WAF after extraction was represented by saturated (about 60%) and polyaromatic (about 20%) hydrocarbons.

The composition of the latter was dominated by low-molecular weight PAHs, such as naphthalene, fluorene, methylanthracene and phenylanthracene. In order for the concentrations of petroleum hydrocarbons that passed into the water phase to approximately correspond to the concentrations that were observed in the water column during oil spills [5,36], the original WAF-DF was subjected to a series of dilutions. Thus, we used concentrations of WAF-DF, which are widely used in ecotoxicological experiments to detect sperm and embryo toxicity [3,11].

Various species of sea urchins show similar sensitivity to the effects of diesel fuel. Thus, the effective EC50 concentrations of diesel fuel for sea urchin larvae were in the dilution range of the initial extract of 27–54% [37]. For the sea urchin *Hemicentrotus pulcherrimus* in 48- and 72-h acute experiments, the EC50 of the WGF of diesel fuel was 3.39 and 1.87 mg/L, respectively [38]. The dilution of marine diesel fuel solution corresponding to EC50 for *Paracentrotus lividus* larvae was 45% [31]. However, the effects of such exposure at the molecular level are still poorly studied.

**Figure 1:**
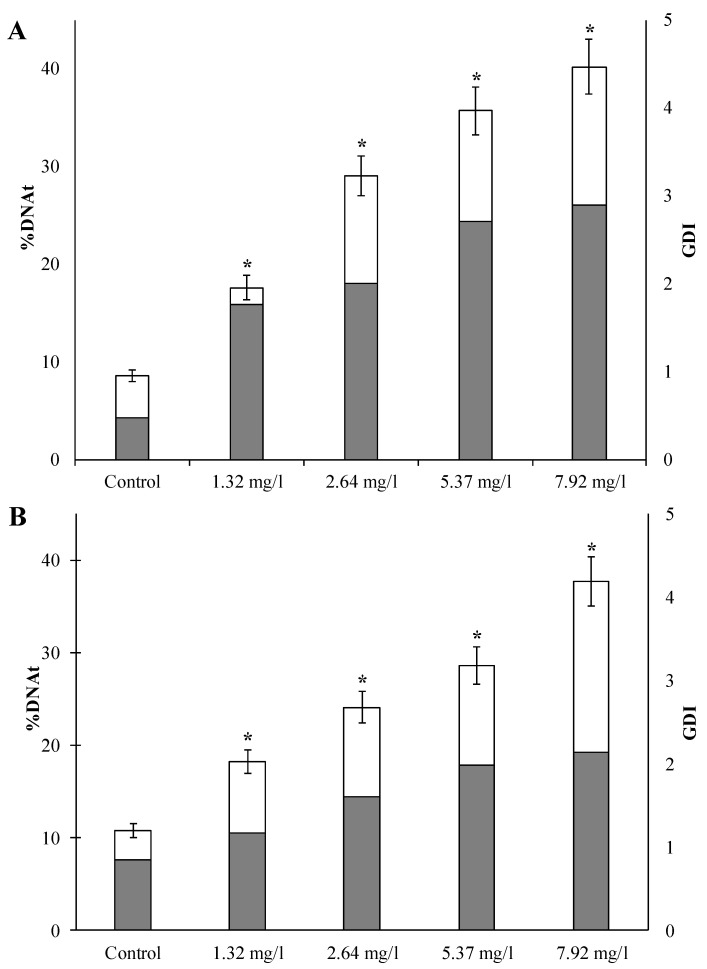
Level of DNA damage in sperm (**A**) and eggs (**B**) of sand dollar *S. mirabilis* after exposure to different concentrations of WAF-DF (mean ± standard deviation). * Difference from the control is significant (*p* < 0.05).

Among all the major cellular components that can be damaged by WAF hydrocarbons, nuclear DNA is a critical target. To determine genotoxicity, we used the Comet assay method in our work, which made it possible to identify the level of genome damage in spermatozoa and eggs of the sand dollar *S. mirabilis* before and after short-term exposure to various concentrations of WAF-DF (Figure 1). The presence of nuclear DNA fragmentation in both untreated gamete species can be explained by the accumulation of alkali-labile regions and/or single- and double-strand breaks during oogenesis [39,40].

According to the experimental data shown in Figure 1A,B, short-term exposure to all studied concentrations of WAF-DF in both types of sand dollars gametes revealed a statistically significant increase in the percentage of fragmented DNA migrating from the nucleus to the tail of the comet, compared with control values.

Common to all experimental groups is a significant increase in the average level of damaged DNA in the “tail” of comets, depending on the concentration of WAF-DF. It is of particular interest that even when exposed to the minimum concentration of WAF-DF (1.32 mg/L), more than 15% of the genome of both sand dollars’ gamete species was damaged.

**Figure 2:**
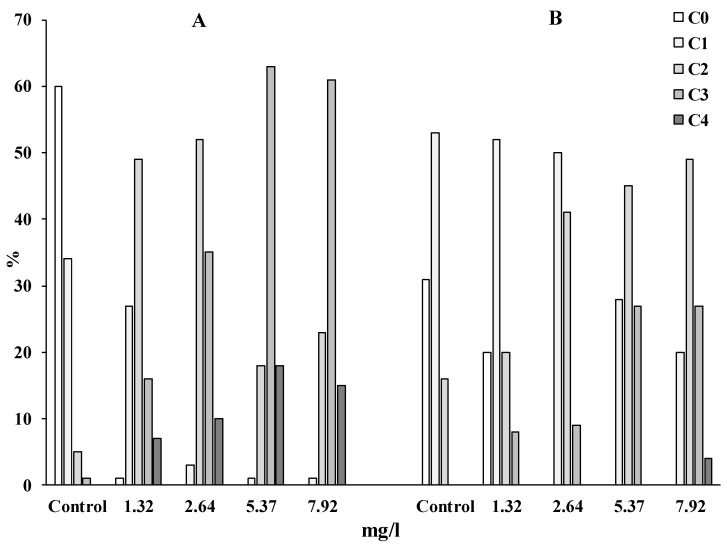
Distribution of comets according to the classes in sperm (**A**) and eggs (**B**) of the sand dollar *S. mirabilis* after exposure to different concentrations of WAF-DF.

For clarity, the experimental data obtained were presented in the form of a diagram (Figure 2), which characterizes the distribution of cells according to the degree of nuclear DNA damage according to the classification [41].

It should be noted that the eggs and spermatozoa of control animals predominantly (85% and 95%, respectively) form comets that belong to the C0 and C1 classes, which are characteristic of intact and slightly damaged viable cells. When exposed to WAF-DF on both types of gametes, comets are formed, predominantly belonging to classes C2, C3 and C4, which indicates a high level of fragmentation of the DNA molecule (Figure 2). This trend is especially pronounced in experiments with sand dollar sperm (Figure 2A). If, in the control groups of sand dollars, sperm with the maximum DNA content in the comet tail (>20%) were no more than 5% of the cells, then after a short exposure to even the minimum concentration of WAF-DF, the proportion of cells with a damaged genome (C2 + C3 + C4) is about 70%. At the same time, it should be noted that with an increase in the concentration of WAF-DF, the number of cells that form comets (C4) of apoptotic nature increases.

At all concentrations of WAF-DF, the values of GDI in both types of gametes exceeded the control level. In experimental gametes, an increase in the GDI values is observed with an increase in the concentration of the active toxicant. It should be noted that at the maximum concentration of WAF-DF used, the GDI value in sperm reached 3, while in eggs it was about 2. Nevertheless, in both cases, these GDI values indicate a very pronounced genotoxic effect of the studied toxicant.

Studies using the comet assay have shown that compounds present in the WAF of petroleum products induce a high degree of DNA damage in the somatic cells of aquatic organisms [5,6,9,11,42]. An interesting example is the work of Baussant et al. [43], in which the genotoxic effect of petroleum hydrocarbons on the early development of hydrobionts was evaluated in chronic experiments. It was shown that after a seven-month exposure of adult mussels to a solution of crude oil at a concentration of 0.25 mg/L, their offspring (embryos at the age of 1 day) accumulated DNA strand breaks. When studying the toxicity of individual components of oil and diesel fuel, it was shown that benzo/a/pyrene induces DNA strand breaks and causes deformities in oyster embryos [44].

At the same time, quite convincing data have been obtained on the high sensitivity of sperm of aquatic organisms to damage by substances exhibiting genotoxic properties [7,18,45]. An increase in DNA damage in sperm in fish and crustaceans after exposure to methyl methanesulfonate has been noted using comet analysis [17,18,46]. Zhou et al. [47] revealed a duroquinone-mediated DNA damage in the sperm of carp. In addition, DNA damage in sperm was observed in oysters (*Crassostrea gigas*) exposed to the pesticide diuron [18,45,48], and after studying the germ cells of *Gammarus fossarium*, it was found that sperm are more sensitive to certain toxic agents than hemocytes. The results obtained by Chatel et al. [49] showed a significant increase in DNA damage in the sperm of estuary bivalve molluscs (*Scrobicularia plana*) exposed to benzo/a/pyrene. Given the high sensitivity of *Palaemon serratus* spermatozoa to genotoxicants, Erraud and colleagues [20] proposed using them as a useful tool for monitoring global marine pollution.

Most researchers, when explaining the causes of genotoxicity, pay attention to the ability of oil hydrocarbons, especially polycyclic ones, to induce an increased generation of reactive oxygen species (ROS), thereby causing oxidative stress. It has been shown that PAHs, accumulating in the cells of planktonic organisms, caused to the ROS generation, which leads to oxidative stress and the formation of intermediate toxic products induced by the cytochrome P-450 monooxygenase system [3]. On cell cultures of mouse fibroblasts, it was also shown that PAHs induced the ROS generation, which caused a DNA double-strand break formation [50]. Oxyradicals are thought to be the main cause of oxidative and DNA damage. In this connection, it can be assumed that it is PAHs (which account for 20%) that are the most toxic compounds for DNA.

It is known that low-molecular weight mono- and diaromatic compounds, such as naphthalene phenanthrene and anthracene, found in WAF-DF, can induce an increased level of superoxide anion (•O^2−^) and cause oxidative and genotoxic effects [5,51,52].

It can be assumed that WAF hydrocarbons, dissolving in the lipid matrix of the outer membrane of gametes and penetrating into the cell, can to some extent disorganize the receptor-signaling system and induce the formation of ROS. Sperm, unlike somatic cells, are potentially more susceptible to damage by substances exhibiting genotoxic properties, since they are practically devoid of the ability to repair DNA and have weak antioxidant protection [7,17,18,45]. As shown earlier, sea urchin sperm are capable of generating at least two types of ROS: H_2_O_2_ and O^2−^ [53]. Additionally, given the relatively hydrophobic nature of WAF-DF, the direct influence of hydrocarbons on the genome and gene structures and/or the DNA repair system cannot be excluded [3].

**Figure 3:**
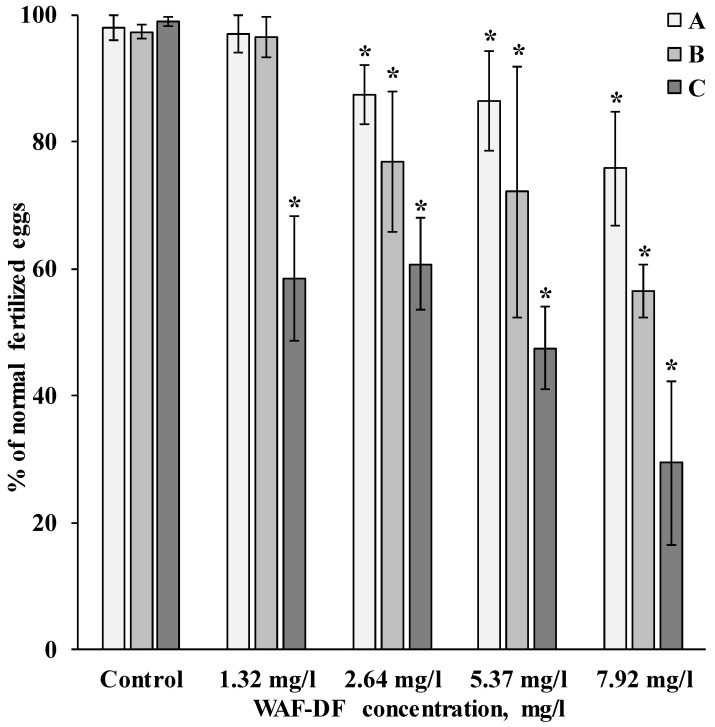
Effect of WAF-DF exposure of different kinds of germ cells on the fertilization of the sand dollar *S. mirabilis* (A—sperm were exposed to WAF-DF; B—eggs were exposed to WAF-DF; and C—both types of gametes were exposed to WAF-DF (mean ±standard deviation). * Difference from the control is significant (*p* < 0.05).

To evaluate the functional properties of gametes with varying degrees of genome damage, we tested their ability to fertilize. The results of the tests performed showed that the effect of WAF-DF exposure depends not only on its concentration, but also on the characteristics of the biological material (Figure 3). In all experiments, there was a relationship between an increase in the concentration of hydrocarbons in the tested solutions and a decrease in the level of fertilization. When treating gametes separately with hydrocarbons, it was shown that eggs are more sensitive to toxic effects.

In oocyte exposure, a sharp decrease in fertilization rates (up to 77%) was observed at a minimum WAF-DF concentration of 2.6 mg/L. After sperm exposure, a decrease in fertilization success was observed at low concentrations of WAF-DF, but the lowest fertilization rate was detected at a hydrocarbon concentration of 7.9 mg/L. When both gametes were exposed to WAF-DF, the fertilization rate at all concentrations was significantly different from the control (Figure 3).

Previously, it was shown that polyaromatic hydrocarbons, which are the main components of petroleum products, have a significant embryotoxic effect on the marine environment [54]. A group of researchers led by J. Bellas [55] calculated the lowest effective concentrations (LOEC) and EC50 for the sea urchin *P. lividus*; this amounted to naphthalene 0.95 mg/L and 4.35–4.48 mg/L, fluorene 6.95 ng/L and 1.98 mg/L, and phenanthrene 0.32–1.3 ng/L and 0.43 mg/L, respectively. The listed EC50 levels of PAHs are close to their content in our dilutions of WAF-DF with high concentrations, which causes their significant embryotoxic effect.

Our results show that sand dollar spermatozoa retained the ability to fertilize eggs with an efficiency of up to 97%, with DNA damage of up to 32–35% (Figure 3). At the same time, in experimental groups of sperm with a level of DNA damage exceeding this range, there was a decrease in the success of fertilization. Moreover, in these experimental groups of sperm, there is a maximum number of cells with a high level of damage, corresponding to the C4 class (Figure 3). Sand dollar eggs were found to be more sensitive to WAF-DF. Decreased fertilization success was observed after exposure of oocytes to 2.6 mg/L WGF-DT, with genome damage averaging about 20%. It should be noted that among the eggs, there were no cells that form C4 class comets; comets corresponding to C3 class accounted for no more than 8–10% (Figure 2).

Based on the results obtained, it is logical to assume that the level of genome damage in eggs and spermatozoa has a threshold value, above which biochemical changes are induced in gametes that disrupt the success of fertilization.

This threshold value, which reflects the sensitivity of the genome to toxicants, is significantly lower for eggs than for sperm. It is possible that the threshold level in DNA destruction affecting fertilization may explain the controversy in identifying the relationship between DNA damage and fertilization success. In the experiments of a number of authors, it is noted that, despite serious damage to the DNA of spermatozoa, the success of fertilization remained at a high level in fish [17,46], the mussel *Mytilus edulis* and the polychaete *Arenicola marina* [48,56]. Nahon et al. [57] showed that radiation damaged the chromatin of the eggs of sea urchins *P. lividus* and *Sphaerechinus granularis*, though despite this damage, the eggs retained the ability to fertilize. Lewis et al. [7] showed that a decrease in fertilization success occurs when exposed to oil and individual PAHs (pyrene, fluoranthene) on the gametes of the polychaetes *A. marina* and *Nereis virens*, in concentrations observed during oil spills.

Sperm and eggs perform the unique and essential biological function of forming a common genome for the development of the next generation. Therefore, the integrity of gamete genomes is of paramount importance for the development of viable offspring. Sperm are highly specialized cells that carry out the transfer of paternal DNA to the egg. Sperm contain highly condensed DNA, have weak antioxidant protection and practically lack the ability to repair DNA damage, and are therefore vulnerable to oxidative stress [20,58]. The induction of DNA damage in sperm cells indicates the inability of the defense mechanisms of sea urchin sperm to protect DNA from the effects of genotoxicants. Accordingly, unrepaired DNA damage—even in the case of successful fertilization—is transferred to the zygotes, participating in the formation of the next generation genome [58,59].

## 4. Conclusions

Our results demonstrate that the genetic material of the sand dollar *S. mirabilis* gametes (oocytes and sperm) is sensitive to the effects of WAF-DF. This indicates that during oil spills, soluble hydrocarbons pass into the water phase in concentrations sufficient to cause damage to the DNA of planktonic organisms. It should be emphasized that testing for the genotoxicity of sea water using the DNA comet method can be not only a diagnostic, but also a prognostic tool for assessing the consequences of an oil spill on the life of coastal organisms. In order to fully identify and evaluate the consequences of exposure to WAF oil products on male and female gametes, longer experiments are needed, including later stages of development. In addition, an assessment of ROS formation is needed in future studies to understand the specific mechanisms of WAF’s toxic effects.

## Figures and Tables

**Table 1:** Qualitative and quantitative composition of hydrocarbons (WAF).

Hydrocarbons	% of the Total	Concentration (mg/L)
Aliphatic hydrocarbons Saturated (C8–C30) Unsaturated	60.97 59.05 1.92	6.45 6.25 0.20
Cyclic hydrocarbons	8.27	0.87
Monocyclic aromatic hydrocarbons	3.87	0.41
Phenols	0.5	0.05
Polyaromatic hydrocarbons (PAHs) Naphthalene and alkylnaphthalene Fluorene and methylfluorene Methylanthracene Phenantrene Methylazulene	19.45 13.27 2.19 1.62 1.27 1.1	2.06 1.40 0.23 0.17 0.13 0.12
Unidentified	6.94	0.73

## Data Availability

Not applicable.
